# Supplementary material for: H3K9me3 is required for inheritance of small RNAs that target a unique subset of newly evolved genes
Source: eLife. 2019 Mar 14;8:e40448. doi: 10.7554/eLife.40448 (PMC6417860; doi:10.7554/eLife.40448)
Supplement: Supplementary file 1. [file elife-40448-supp1.docx]

>anti-*gfp* RNAi sequence

GTGCTGAAGTCAAGTTTGAAGGTGATACCCTTGTTAATAGAATCGAGTTAAAAGGTATTGATTTTAAAGAAGATGGAAACATTCTTGGACACAAATTGGAATACAACTATAACTCACACAATGTATACATCATGGCAGACAAACAAAAGAATGGAATCAAAGTTAACTTCAAAATTAGACACAACATTGAAGATGGAAGCGTTCAACTAGCAGACCATTATCAACAAAATACTCCAATTGGCGATGGCCCTGTCCTTTTACCAGACAACCATTACCTGTCCACACAATCTGCCCTTTCGAAAGATCCCAACGAAAAGAGAGACCACATGGTCCTTCTTGAGTTTGTAACAGCTGCTGGGATTACACATGGCATGGATGAACTATACAAA
